# Supplementary material for: The endoplasmic reticulum stress-autophagy pathway controls hypothalamic development and energy balance regulation in leptin-deficient neonates
Source: Nat Commun. 2020 Apr 20;11:1914. doi: 10.1038/s41467-020-15624-y (PMC7171135; doi:10.1038/s41467-020-15624-y)
Supplement: Supplementary file 1 — Supplementary Figures [file 41467_2020_15624_MOESM1_ESM.pdf]

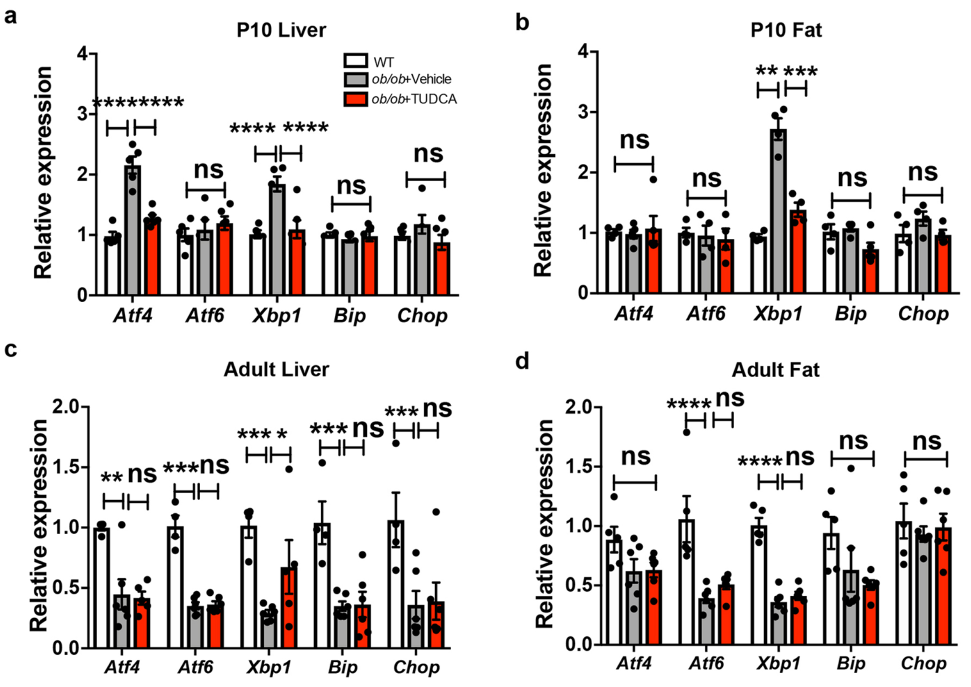


**Supplementary Fig. 1. ER stress markers in peripheral tissues. a-d** Relative expression of *Atf4, Atf6, Xbp1, Bip*, and *Chop* mRNA in **(a, c)** liver and **(b, d)** adipose tissue of **(a, b)** postnatal day (P)10 and **(c, d)** 10-week-old wild-type (WT) mice and *ob/ob* mice treated neonatally with vehicle or tauroursodeoxycholic acid (TUDCA) (n = 6 per group). Error bars represent the SEM. *$P \leq 0.05$, **$P < 0.01$, ***$P \leq 0.001$, and ****$P \leq 0.0001$ *versus* all groups. Statistical significance was determined using two-way ANOVA followed by Tukey's multiple comparison test **(a-d)**. Source data are provided as a Source Data file.

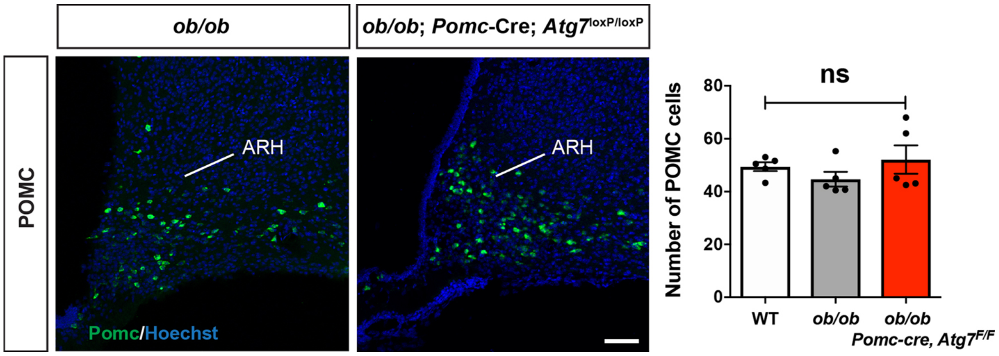


**Supplementary Fig. 2. Leptin and autophagy deficiency does not affect *Pomc* cell numbers.** Representative image and quantification of the number of *Pomc* mRNA-expressing neurons (*green fluorescence*) in the arcuate nucleus (ARH) of 10-week-old wild-type (WT), *ob/ob*, and *ob/ob; Pomc*-Cre*; Atg7*$^{loxP/loxP}$ mice (n = 5 per group). Error bars represent the SEM. Statistical significance was determined by one-way ANOVA followed by Tukey's multiple comparison test. Scale bar, 50 μm. Source data are provided as a Source Data file.

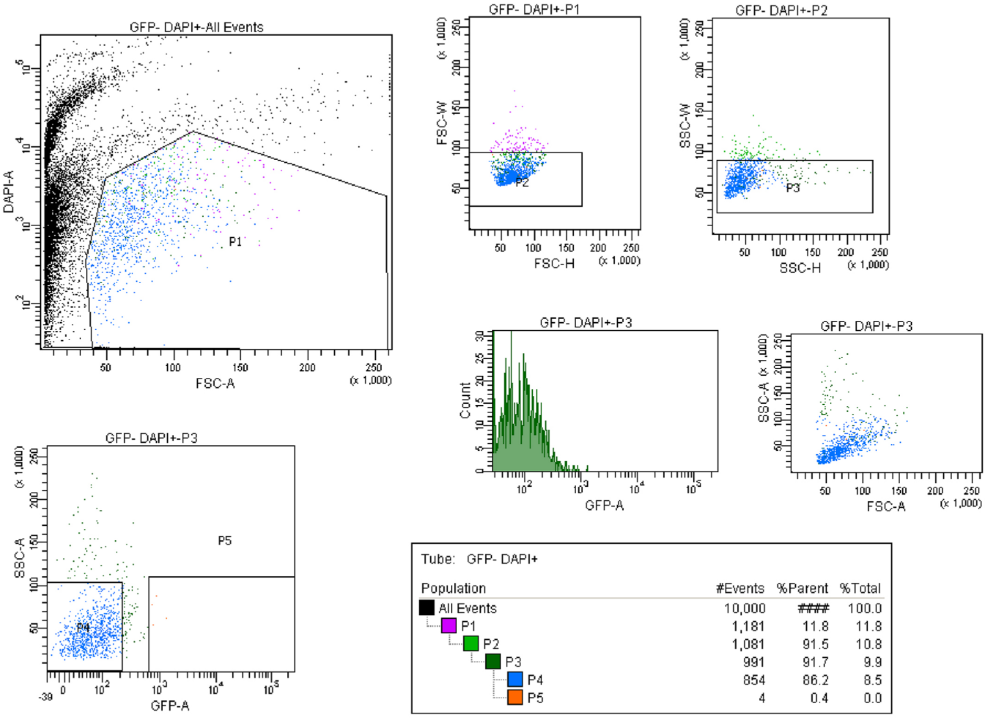


**Supplementary Fig. 3. Representative gating strategy.** Gating strategy used to analyze *Pomc*-GFP+ cells, which corresponds to FACS data panel in Figure 5d.

| Primer name | Sequence (5’ to 3’) |
|---|---|
| ob/ob forward | CGT GCA GTC TAT CAA CAG GTC C |
| ob/ob reverse | TGT GGA GTA GAG TGA GGC TTC C |
| Atg7 forward | TGG CTG CTA CTT CTG CAA TGA TGT |
| Atg7 reverse | CAG GAC AGA GAC CAT CAG CTC CAC |
| Pomc-Cre forward | GCC CTG GAA GGG ATT TTT GAA GCA |
| Pomc-Cre reverse | ATG GCT AAT CGC CAT CTT CCA GCA |
| LC3 forward | TCC TGC TGG AGT TCG TGA CCG |
| LC3 reverse | TTG CGA ATT CTC AGC CGT CTT CAT CTC TCT CGC |

**Supplementary Table 1. Primers used for mouse genotyping**
